# Analysis of Factors Affecting Academic Performance of Mathematics Education Doctoral Students: A Structural Equation Modeling Approach

**DOI:** 10.3390/ijerph20054518

**Published:** 2023-03-03

**Authors:** Tommy Tanu Wijaya, Boran Yu, Fei Xu, Zhiqiang Yuan, Mailizar Mailizar

**Affiliations:** 1 School of Mathematical Sciences, Beijing Normal University, Beijing 100875, China; 2 Teachers College, Columbia University, New York, NY 10027, USA; 3 School of Mathematics and Statistics, Hunan Normal University, Changsha 410081, China; 4 Mathematics Education Department, Universitas Syiah Kuala, Banda Aceh 23111, Indonesia

**Keywords:** mathematics education doctoral students, academic performance, stress level, well-being, PLS-SEM

## Abstract

Student academic performance is an important indicator of doctoral education quality, but limited research has focused on how multiple influential factors of doctoral students’ academic performance work together. This study aims to explore the factors significantly affecting the academic performance of mathematics education doctoral students in Indonesia. Several factors were recognized from prior studies, such as the fear of delay, student engagement, parental support, teacher support, facilitating conditions, stress level, and well-being. An online questionnaire was designed and answered by a total of 147 mathematics education doctoral students. The partial least squares structural equation modeling (PLS-SEM) approach was adopted to analyze the questionnaire data. The results suggested that teacher support had the strongest positive effects on mathematics education doctoral students’ academic performance in Indonesia. Student engagement was the most significant positive factor in improving doctoral students’ well-being, while parental support could most significantly reduce their stress levels. Practically, these results are expected to provide implications to universities and supervisors regarding the improvement of doctoral students’ well-being to promote their academic success and further the quality of doctoral programs in education. Theoretically, these results can also contribute to building an empirical model that can be used to explore and explain how multiple factors could affect doctoral students’ academic performance in other contexts.

## 1. Introduction

Achieving academic success at the doctoral level is very arduous, and there is a high dropout rate and a poor satisfaction level among doctorate students [1,2]. The doctoral attrition rate varies from 30% to 90% across different cultural backgrounds [3,4], and the average satisfaction rate of doctoral programs was not optimistic [5]. Attrition of doctoral students indicates a high price for institutions, as the Ph.D. students are mostly responsible for a large share of academic output and financial input [6]. More importantly, Ph.D. students, who were admitted to the doctoral programs with the most brilliant achievement, suffered from the heavy cost of dropout. Though quitting doctoral programs does not mean failure, students may suffer from physical and psychological disorders as well as a great loss of time and money [7].

Contrary to the high dropout rate of doctoral students, previous studies suggested a proliferation of doctoral education programs in East Asia. Policymakers started to provide equally intensive input into doctoral education as their counterparts in America and Europe to increase the number of world-class ranked universities [5]. With a strong intent of training domestic scholars and knowledge-makers to improve university research performance, active reforms were implemented to improve doctoral training quality and raise the number of doctorates within East Asian countries. Examples include Brain Korea 21, China’s 985 Project, and Japan’s Top Global University. According to UNESCO, China has become the world’s second-largest doctoral-degree-granting system. Other developing Asian countries also witnessed a rapid increase in the number of students involved in doctoral programs [8,9,10]. However, most studies on East Asian doctoral education concentrated on developed countries, with less emphasis on other developing nations. Considering this situation, the exploration of the factors affecting the academic achievement of doctoral students is of great practical significance for empirical evidence and implications on how to provide better support for Ph.D. students in developing East Asian countries.

The latest trend in the investigation of doctoral students’ academic success is to stress the importance of individual, social, and environmental factors. Scholars have studied multiple psychological factors at the individual level that can influence doctoral students’ achievement. As individuals’ consistent endeavors are indispensable to generate academic output, students’ willingness to become engaged in learning was well recognized as a significant indicator of their academic performance in the higher education context [11,12]. The findings on student engagement have triggered researchers’ interest in other emotional factors that can influence the extent to which students actively get involved in their doctoral training. Besides the well-studied importance of doctoral students’ well-being as a premise for active learning [13], negative emotions such as fear and stress were suggested to have unfavorable effects on their academic performance. Pascoe and colleagues examined the impact of stress across different educational levels and found that stress can undermine students’ learning capacity, bring mental and physical health issues (e.g., depression and sleep disturbances), and further influence their academic performance [14]. Such stress can be more common among social sciences doctoral students as they will find it more difficult to secure financial support than their peers in nature sciences [15]. Similarly, the fear of failure can increase doctoral students’ mental health problems and have further negative effects on their academic achievement [16]. Such fear is more common for doctoral students in social sciences as they tend to spend more time to obtain degrees than their peers in natural sciences or humanities [2,17,18]. Besides the significance of these individual factors, it is also believed that support from parents, supervisors, and institutions are of equal importance in completing doctoral degrees [19,20,21]. These studies lamented insufficient exploration of how individual and environmental factors can work together in doctoral students’ academic success in a specific area. Therefore, it is necessary to explore the complex mechanism of factors contributing to doctoral students’ success in a specific branch of social sciences.

Previous studies examining the influential factors of doctoral students’ success rarely focused on the multifaceted nature of achievement at the doctoral level. The completion of the doctoral program was commonly used as an indicator of academic success at the doctoral level. However, according to Bagaka’s et al. [22], taking “All But Dissertation” (ABD) as equivalent to the success of doctoral students undermined the aim of successful doctoral education programs. To prepare scholars to be sufficiently grounded in subject content and research, doctoral education programs have more ambitious goals of individual achievement, including performance in coursework, mastery of subject content knowledge, academic writing and oral communication skills, self-learning ability, and critical thinking ability, as well as valuable personal characteristics such as perseverance and commitment to excellence. According to Cullen, subjects in social science are identified to belong to the Pure/Applied cluster, where there is no specific paradigm but a concentration on practice in the subject [23]. Studies focusing on social science doctoral students’ academic achievement have specified many skills that marked a successful doctoral student (e.g., academic writing skills [24], learning strategies [25], and a synthesis of academic skills and coursework [26]). In the field of education, one of the most practice-oriented social science subjects, there are more requirements to prepare qualified education doctoral students (e.g., their epistemological diversity [27], and their ability as a practitioner researcher [28]). Though there were many studies on education doctoral students, few quantitative studies focused on the academic performance of mathematics education doctoral students as a specialization field in education.

Doctoral programs in mathematics education were provided worldwide to prepare mathematics educators with doctorates [29,30,31]. The U.S. had the first mathematics education doctoral programs in the 1900s, and the number of people with a doctorate in mathematics education is increasing [32]. Countries in Asia have also witnessed an increase in the number of mathematics education doctoral graduates, though this number is always limited (e.g., twenty a year in China). The Indonesian government has been making efforts to increase the number of researchers with doctorates in mathematics education. In recent years, each Indonesian university has admitted 15–20 doctoral students in mathematics education every year. The length of doctoral programs in Indonesia is 3–4 years, and students generally need a master’s degree in a relevant major and proficiency in at least one foreign language, such as English or Mandarin to obtain admission to the program. The students should also have good analytical skills and the ability to collaborate with teams. Moreover, every doctoral student needs to obtain at least 72 credits and satisfy the requirement of research activities for graduation. These activities include assistantship in research projects, presentations at international conferences, and at least 2 Scopus-indexed journal publications. Mathematics education doctoral students in Indonesia are required to report to the supervisor regarding proposal writing, the progress of data collection, and the presentation of results. Before advancement to graduation, the research outputs and dissertations need to be reviewed by the academic committee, which consists of three professors in mathematics education. Most of the mathematics education doctoral students in Indonesia are married, and some of them are employed by universities or government agencies with tight schedules. A few of them graduated in three years, but on average it took 4 to 5 years to finish their programs. However, funding is provided for four years at most. In this case, the main problems commonly encountered by these students are expensive tuition fees if they are delayed in achieving the doctoral degree, and publications in Scopus-indexed journals.

This study aims to explore and determine the factors affecting the academic performance of mathematics education doctoral students in a developing Asian country, as well as investigate the underlying mechanism. Based on the questionnaire data obtained from 147 Indonesian doctoral students in mathematics education, the structural equation modeling approach was used to explore how the pre-identified factors can affect their academic achievement. This statistical evidence would suggest the power of different influential factors, as well as be useful in the construction of their interactive paths toward influencing academic success at the doctoral level. Moreover, the results obtained are expected to contribute to the theoretical framework of doctoral students’ academic success and its link with multiple personal and environmental factors. These contributions could provide practical implications for stakeholders regarding how to better support mathematics education doctoral students in their academic careers.

## 2. Theoretical Background and Hypothesis Development

In this section, we first specify indicators of doctoral academic performance. It follows by reviewing prior studies on factors affecting doctoral students’ academic performance. The review results are synthesized according to the seven individual and environmental factors we identified from past studies. Finally, we developed our hypotheses to build the empirical model of influential factors.

### 2.1. Doctoral Students’ Academic Performance Indicators

Great efforts have been made by higher education studies to investigate the quality of doctoral education and the academic performance of Ph.D. students. Recent research adopted the rates of attrition [33], retention [34], completion [35], or on-time graduation [20] as indicators of doctoral education quality. These indicators separately portrayed the academic achievement of doctoral students based on the following two perspectives: continuous enrollment in their doctoral study and completion of their degrees (in time). For doctoral success, the use of these indicators is rational, considering the high dropout rates of doctoral students across different cultural backgrounds. However, Olehnovica et al. [36] suggested that the success of doctoral study should be more than “all but dissertation” (ABD) and be highly concerned with the competencies of doctorates as a scholar, including knowledge of research in specific areas and critical interpersonal skills [36]. Although obtaining a doctoral degree is counted as a great achievement, key indicators of doctoral students’ achievement also include performance in coursework, mastery over subject content knowledge, academic writing and oral communication skills, self-learning ability, critical thinking ability, as well as valuable personal characteristics (e.g., perseverance and commitment to excellence) [37]. These studies have highlighted doctoral success as a combination of internal and external achievement, despite the huge differences in the academic performance of doctoral students across disciplines.

Multiple indicators were used to comprehensively delineate doctoral students’ academic performance rather than simply measuring their completion of degrees. In the field of education, one of the most practice-oriented subjects, there are more requirements to prepare education doctoral students (e.g., epistemological diversity [27] and the ability as a practitioner researcher [28]). On the basis of literature and theory review, Olehnovica and colleagues [36] divided the academic competence of the students into three categories according to the context and the level of comprehension. The first one is informative competence (F1), which concerns decisions on the structure, content, and strategy of research. Secondly, communicative competence (F2) refers to the ability to become involved in local and global research environments. The last indicator is instrumental competence (F3), which concerns collection procedures and interpretations of the research data. The comprehensive framework they proposed to specify doctoral students’ competence was proven to be valid among students who participated in the “Pedagogy” doctoral program. This validation also justifies our reference to this framework to conceptualize mathematics education doctoral students’ academic performance.

### 2.2. Factors Affecting Doctoral Students’ Academic Performance

Previous studies have explored multiple factors affecting the academic achievement of doctoral students, and exposed the multifaceted nature of doctoral success [4,21,33,38]. According to Leijen and colleagues [38], the factors contributing to the progress of doctoral students can be categorized into three groups: personal characteristics, supervisory arrangements, and the wider learning community. As the nature of the doctoral study is an arduous process, individuals’ efforts, physical health, and psychological well-being were the premises of their success [7]. Castelló et al. [4] investigated the institutional and personal variables influencing the dropout of doctoral students. Besides the significance of support from supervisors, the family was proven as another important source of social support that positively affects doctoral students’ academic achievement. Support from family is more important in East Asian countries, whose students had high levels of family support [39]. Such support is more necessary for education Ph.D. students, as they may face greater difficulties in receiving funding than their peers in nature sciences [15]. In this study, we focused on several predictors found in previous research, including fear of delay, student engagement, parental support, teacher support, facilitating conditions, stress level, and well-being. These factors will be comprehensively explained in the following sections.

### 2.3. Fear of Delay

Fear is a basic human emotion that may cause negative consequences, for example, academic failure. It could also lead to stress, excessive anxiety, and other psychological or physical disorders [40]. According to previous studies, fear negatively influenced academic performance and was linked to poor performance during examinations [40,41,42]. However, other studies emphasized the positive effects of fear, such as motivating efforts and self-introspection [43]. In this case, fear as the feeling of being afraid of academic failure encouraged students’ effort to achieve goals. In this study, doctoral students in Indonesia were, generally required to complete their degrees within a maximum of 5 years, with scholarships guaranteed for no more than 4 years. Since most students were married, and some of them employed, they felt the duration was too short for fulfilling doctoral study. In this case, a delay in doctoral academic progress may bring detrimental effects on their career, finance, and family. Therefore, the fear of delay is supposed to significantly increase their stress levels, and, further, significantly impose a negative influence on their academic performance.

### 2.4. Student Engagement

Student engagement is the willingness to invest time, energy, and intellectual efforts in learning activities [44,45]. Student engagement can be divided into three categories, namely behavioral, emotional, and cognitive [46,47]. It was recognized as an important factor that catalyzes students’ efforts and further contributes to students’ learning and achievement across different educational levels [48,49,50]. Student engagement was also found to be related to their well-being [45,46,49,51]. In this study, student engagement is defined as the willingness of mathematics education doctoral students to engage in doctoral learning activities such as reviewing the literature, seeking research opportunities, collaborating with colleagues, developing research instruments, discussing with supervisors, conducting studies, and publishing results. This definition leads to the prediction that mathematics education doctoral students’ academic performance can be influenced directly by student engagement. In addition, higher engagement is predicted to reduce their stress levels.

### 2.5. Parental Support

In past studies, parental support was often proven to be positively associated with students’ academic achievement and was identified as an important factor in academic success [41,52]. Strong support from parents commonly has positive effects on motivation for learning, involvement in education [53], and student attitudes toward learning [54]. Such support can also be divided into two categories, namely, academic and emotional support.

Results from studies that focused on the effects of paternal support on students’ academic achievement were inconsistent. A recent meta-analysis study suggested that parental support has a significant positive effect on student achievement [55]. However, another study showed that over-supportive families in East Asian countries were not significantly associated with educational outcomes [56]. From this result, parental support did not always significantly influence learning progress. In this study, parental support is predicted to significantly reduce stress levels and increase the academic performance of mathematics education doctoral students.

### 2.6. Teacher Support

Support from parents and teachers are considered of equal importance in students’ learning [57]. Previous research suggested a positive relationship between teacher support and students’ academic performance at different educational levels [19,58]. This positive relationship was also found at the doctoral level, and the supervisor (or advisor) was proven to be critical in doctoral students’ mental health status and academic performance [2,18,20]. Considering the importance of collaboration skills in the 21st century, teacher support also motivates and facilitates doctoral students’ opportunities to become involved in the research community [59,60,61]. The opportunities for learning from leading scholars in the field can significantly improve the research ability of doctoral students. Indonesian teachers are always considered as an important source of knowledge for students when they encounter educational difficulties. In this study, teacher support is defined as the in-time assistance provided by supervisors and other institutional members to all mathematics education doctoral students. Therefore, teacher support is predicted to influence the academic performance and well-being of mathematics education doctoral students in a significantly positive way.

### 2.7. Facilitating Conditions

Facilitating conditions are conditions where mathematics education doctoral students pursue a degree and discuss research problems with their peers. In this case, the institution needs to provide necessary educational facilities, such as seminars and training, to improve the research abilities of doctoral students. It has been shown that facilitating conditions influenced individual behaviors [57,62,63], indicating the factor indirectly affected student academic achievement during a pandemic [64]. For our analysis, facilitating conditions are predicted to positively influence student well-being and effectively improve their academic performance.

### 2.8. Stress Level

Stress is a feeling of emotional or physical tension, and it is common among doctoral students who usually face numerous difficult tasks [44,65]. This factor was found to negatively influence the psychological health of students, and thus attracted several scholars to consider and assess its relationship to academic performance [66,67]. Interestingly, several analyses found that stress is rarely beneficial for individuals [41,68]. This is different from other studies, where stress was found to commonly motivated people to learn and improve their academic performance. It was also suggested that strategies to deal with stress are likely to become essential skills for future citizens. Therefore, this study predicted that stress level has a significant negative effect on the academic performance of mathematics education doctoral students. It is also predicted that parental support and student engagement are likely to reduce the stress level of doctoral students.

### 2.9. Well-Being

Students’ well-being affects various aspects of their academic performance, including learning abilities, engagement [69], achievement [49], and teamwork capability [70,71]. Besides relieving students’ mental problems, stress, and frustration, well-being could also inspire doctoral students’ innovative ideas for education research problems [72,73]. From this context, well-being is more defined as the capacity and resources to handle negative emotions, as well as being continuously active and efficient during learning.

Well-being has also been explored as another important indicator of doctoral education quality, except for academic performance [74]. It is believed that maintaining doctoral students’ well-being is an important responsibility for both institutions and supervisors [13,45]. Reducing stress levels and an increase in students’ engagement may increase students’ well-being [49]. On the one hand, overwhelming stress can cause mental health disorders and further affect students’ sleep quality and concentration at work, which can be detrimental to their well-being [41]. On the other hand, students’ increased engagement in the environment can enable productive interactions with peers and supervisors that will bring positive effects on their well-being [49]. Therefore, this factor is predicted to be influenced by facilitating conditions, teacher support, students’ stress level, and their engagement. It is also predicted to be significantly and positively associated with the academic performance of mathematics education doctoral students.

**Figure 1:**
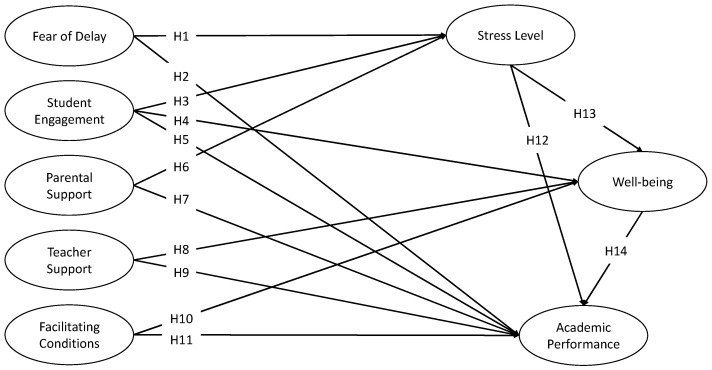
A proposed model for exploring factors affecting mathematics education doctoral students’ academic performance.

Based on the literature review, 14 initial hypotheses containing five independent variables, two intermediated variables, and one dependent variable are displayed in Table 1 and Figure 1.

## 3. Methodology

This study aims to investigate factors that would significantly affect the academic performance of mathematics education doctoral students in Indonesia and how these factors can work together to affect their academic performance. We identified fear of delay, student engagement, parental support, teacher support, facilitating conditions, stress level, and well-being as foci of this study. Based on the empirical model developed from a literature review, we collected data by a self-designed questionnaire. This questionnaire was answered by 147 Indonesian mathematics education doctoral students. We performed quantitative analyses of key elements that affect their academic performance in order to inform stakeholders on how to support doctoral students in their academic careers.

### 3.1. Instrument Development and Data Collection

To obtain more accurate data for our research, we designed an online questionnaire via Google Docs for distribution instead of using publicly available online data. The questionnaire was constructed using a 5-point Likert scale ranging from strongly disagree (1) to strongly agree (5). Additionally, all items in the questionnaire were adapted from previous similar research and modified to fit the context of this study. After the initial design of the questionnaire, and before the questionnaire was distributed to the participants, three experts were invited to review the questionnaire, and changes and refinements were made based on their feedback.

The final version of the questionnaire contained two sections. The first section consisted of the basic demographic information about the doctoral students, including gender, age, marital status, employment, and academic year. The second section focused on the seven pre-identified factors that might contribute to mathematics education students’ academic performance. There were 31 items that focused on the fear of delay, engagement, parental support, teacher support, facilitating conditions, stress level, well-being, and academic performance. These items were adapted from previous research, and the average time required to complete the questionnaire was found to be 9 min. The complete questionnaire with the original sources of items is shown in Appendix A.

Then, the questionnaire was formally distributed to the target group, Ph.D. students majoring in mathematics education, via email, WhatsApp groups, and university professors, in August, September, and October 2022. The validity and reliability of the questionnaires were tested to be good. Also, it was clarified that filling out the questionnaire was voluntary. Participants did not need to fill in their names so that the anonymity of the data could be maintained. The data obtained were analyzed only for the study purpose, without any disclosure. Table 2 showed the detailed demographic data of the participants.

After removing invalid responses, such as incomplete responses and abnormal speed of completing the questionnaire, the final sample consisted of 147 mathematics education doctoral students. A total of 59 (40.14%) participants were male and 88 (59.86%) were female, which is consistent with the result that there are more female Ph.D. students than male Ph.D. students, as indicated by world statistics. The 25–29-year-old students were the highest proportion of all the doctoral students collected, at 58.50%, while 27.89% were 20–24 years old, and 13.61% were 30 years old or older. In addition, the distribution of academic years to which these participants belonged was relatively even, with 15.65%, 27.89%, 35.37%, and 21.08% of students in their first, second, third, and fourth and higher years of doctoral study, respectively. Most of the students were married, at about 60%, while only a very small number of students had already been employed; about 80% of students had not worked yet. Since not many doctoral students were admitted yearly in Indonesia, a total of 147 was considered sufficient to explore the doctoral students majoring in mathematics education.

### 3.2. Data Analysis

We used SPSS 23 and SmartPLS4 to analyze the questionnaire data. Firstly, data cleaning and descriptive analysis were performed by SPSS 23. Then, we decided to use SmartPLS4 to carry out our partial least squares structural equation modeling (PLS-SEM) to study the effects of fear of delay, engagement, parental and teacher support, facilitating conditions, stress level, and well-being on the academic performance of doctoral students.

According to Hair et al., PLS-SEM is a causal-predictive approach to SEM, which overcomes the apparent dichotomy between explanation and prediction by emphasizing prediction in estimating statistical models and having structures designed to provide causal explanations [75]. It performs its estimating function of partial model structures by combining principal components analysis with ordinary least squares regression. Unlike other models that only consider common variance for estimation, PLS-SEM estimates parameters by the total variance, which is referred to as variance-based estimation. Additionally, since the PLS-SEM computes measurement and structural model relationships separately, it succeeds in providing solutions for small sample sizes when the model consists of many constructs and a large number of items. Meanwhile, it is also suitable for analyzing large data sets, including secondary data.

However, as with other multivariate methods, PLS-SEM cannot change sample quality to perform a valid model estimation [75]. Although PLS-SEM results may be affected by nonnormal data under a limited number of situations [76], the robustness of PLS-SEM shown in limited data sets tested as nonnormal contributes to the reason for using PLS-SEM. PLS-SEM also contains high statistical power, which increases the possibility to identify relationships as significant when they are indeed present in the population. Moreover, it has some user-friendly software packages that can be directly and easily used in software environments such as R. Additionally, the evaluation of PLS-SEM results starts with the examination of the measurement models, and the relevant criteria can vary according to the construct type—reflective or formative—and once the measurement models meet all the criteria, the next step is to assess the structural model. The rules of thumb that serve as guidelines to evaluate PLS-SEM results vary depending on the context. For example, the minimum reliability for exploratory research is 0.60 while the minimum reliability for research is 0.70. Finally, the researchers have to run one or more robustness checks, depending on the research context, to support the stability of the results.

The PLS-SEM approach is more suitable for predicting and building new empirical models [77]. It was also verified as valid for analyzing and identifying factors on the academic performance of doctoral students and explaining theoretical constructs in complex models [78,79], which are aligned with our research questions. Therefore, we decided to use SmartPLS4 to carry out our partial least squares structural equation modeling (PLS-SEM) to study the effects of fear of delay, student engagement, parental support, teacher support, facilitating conditions, stress level, and well-being on the academic performance of mathematics education doctoral students.

In the specific use of PLS-SEM for this study, we performed two steps. For the first step, we focused on the analysis of the measurement model [75], which was conducted to confirm the reliability and validity of the model, mainly based on CR (composite reliability) and Cronbach alpha values, as well as the estimation of outer loadings and AVE. Then, Fornell–Larcker [80] and HTMT values were assessed for discriminant validity. For the second step, the structural model was evaluated to test the initial hypotheses and draw conclusions. In this case, a bootstrap method with 5000 subsamples was used in the SmartPLS4 to calculate path coefficients, *t*-values, and *p*-values.

## 4. Results

After analyzing the descriptive statistics and applying the measurement model, our results showed that the academic performance of mathematics education doctoral students was directly affected by student engagement, teacher support, and well-being. The results also showed that the fear of delay can largely increase students’ stress levels, while parental support can significantly decrease their stress levels. Additionally, student engagement, teacher support, and facilitating conditions significantly increased the well-being of doctoral students. However, high levels of stress significantly decreased students’ well-being. Furthermore, the structural model analysis and preliminary hypothesis testing were conducted to determine the factors significantly affecting the academic performance of mathematics education doctoral students in Indonesia.

### Analysis Measurement Model

After examining the indicator loadings, assessing internal consistency reliability, and the convergent validity of each construct measure, the results show that all the indicator loading values were higher than 0.708, except AP1 (0. 651) and AP4 (0.694), and the lowest average variance extracted (AVE) value was 0.556, which is higher than 0.50. Hence, according to Hair et al. [75], the outcome indicates that the construct explains more than 50% of the indicator’s variance and thus can prove acceptable reliability and convergent validity. Besides, the Composite Reliability and Cronbach’s Alpha estimations exceeded the 0.60 limits [75]. Detailed results of outer loadings, reliability, and convergent validity are provided in Table 3.

Then, we assessed the discriminant validity by observing the Fornell–Larcker value [80] and calculating the heterotrait–monotrait ratio (HTMT) of correlations, which is the mean value of the item correlations across constructs relative to the (geometric) mean of the average correlations for the items measuring the same construct, and both results showed that the data we used were good enough to pass the discriminant validity testing. For Fornell–Larcker, Table 4 shows the correlation matrix with AVE values, where all bold representations are higher than the squared estimates of the corresponding variables, which means the data passes the discriminant validity testing. For the HTMT, Table 5 shows that the highest HTMT value was 0.864, which indicates that all values are below 0.90, and according to Hair et al. [75], the discriminant validity is ensured and can be declared to be good.

After the satisfaction of the measurement model assessment, we then evaluated the structural model, and here we conducted the examination of collinearity, R^2^ value, and significance of path coefficients. A significance level of 0.05 was also used with subsamples of 5000, according to the development and recommendation of Hair et al. [75].

Based on the collinearity test, the VIF value was analyzed, with Table 6 showing that all the estimations were not higher than 5. This proved that a multicollinearity problem was not found in the PLS-SEM approach.

**Figure 2:**
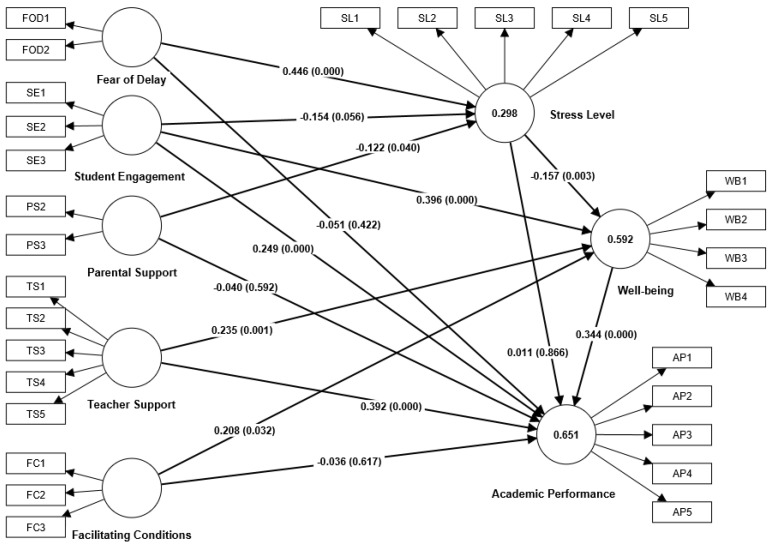
Structural model evaluation results with R^2^, path coefficients, and p values.

Regarding the results, the R^2^ values of academic performance, stress level, and well-being were 0.651, 0.298, and 0.592, respectively (Figure 2). This indicated that more than 65% power was observed and used to explain the factors significantly affecting the academic performance of mathematics education doctoral students, and we can say that it is sufficient to explain the variance within the academic performance of mathematics education doctoral students, as the estimation of the values is higher than 0.1.

Based on Table 7, 9 of the 14 hypotheses were supported. This proved that fear of delay and parental support significantly affected stress levels. Meanwhile, student engagement did not significantly reduce these levels. From the results, student engagement, teacher support, and facilitating conditions significantly influenced the well-being of mathematics education doctoral students. Student engagement, teacher support, and well-being also significantly affected academic performance. The final model with R^2^, path coefficients, and *p* values is shown in Figure 2.

According to these results, student engagement and parental support were the biggest factors significantly influencing well-being and reducing stress levels, respectively. Meanwhile, teacher support was the biggest factor significantly improving the academic performance of mathematics education doctoral students.

Based on these results, fear of delay, parental support, facilitating conditions, and stress level did not directly affect the academic performance of mathematics education doctoral students. This was different from several previous studies, where parental support, stress level, and facilitating conditions significantly and directly influenced individuals. In addition, student engagement, teacher support, and stress level significantly and indirectly affected mathematics education doctoral students’ academic performance (Table 8).

## 5. Discussion

This study is intended to explore the factors that can affect the academic performance of mathematics education doctoral students in Indonesia. We first identified several individual and environmental factors from a literature review, including fear of delay [16], student engagement [22,26], parental support [21,74], teacher support [2,7], and facilitating conditions [38,81]. Then we built a theoretical model of influential factors on doctoral students’ academic performance. The PLS-SEM approach was used to analyze questionnaire data from 147 mathematics education doctoral students and test the theoretical model with hypotheses. Despite the theoretical model being literature-based, our study has suggested findings that are either consistent or inconsistent with past empirical evidence.

Our study suggested that fear of delay could increase mathematics education doctoral students’ stress levels, but could not significantly improve their academic performance. This result is inconsistent with previous studies that revealed the capability of fear of failure and stress in motivating people toward the best goal achievement [42,82]. According to Daniel [83], students’ anxiety increased when fear was experienced, and the increased level of anxiety often motivated individuals toward better performance. However, students often had difficulties concentrating and achieving their best performance when excessive fear and high levels of stress were experienced. This was in line with many previous studies, where people performed better when not under excessive stress and fear [40,41,42]. As doctoral students are always facing a great fear of failure to obtain a doctoral degree, more negative feelings such as fear and stress cannot improve and increase their performance and learning interest. In this case, doctoral students were more able to produce excellent performance when they felt less stressed and learned in a supportive environment. Indonesian mathematics education doctoral students tend to attach great importance to their learning and their high stress levels can impede their achieving maximum learning outcomes. Despite these conditions, supervisors and faculties in Indonesia tend to scare students with the word, “drop out”, as a stimulus to encourage them to complete their studies and achieve their best performance. Results of this study suggested that increasing students’ fear and stress were ineffective and inappropriate to improve the academic performance of doctoral students. The results also suggested that universities should care for the mental well-being of the students, as overwhelming negative feelings such as fear and stress are common in pursuing a doctoral degree. Subsequently, Indonesian doctoral supervisors need to encourage doctoral students in their learning and convince them that the doctoral program can best prepare them for their future.

It is highlighted that student engagement directly and positively affected the academic performance of mathematics education doctoral students. The higher the level of engagement, the better they conceived their achievement in academia. This was in line with previous studies that showed that students, with more enthusiasm for inputting their time and energy into their education, tend to achieve better academic performance [47,84,85]. From this context, institutions and supervisors should endeavor to motivate the engagement of doctoral students majoring in mathematics education. In addition, the statistical results showed that the level of student engagement was capable of significantly increasing their well-being, and indirectly increasing their academic performance, of students pursuing a doctoral degree. This is probably because a strong engagement in academic activities would enable their active involvement and quicken their progress in the doctoral program, which could make them feel more joyful and fulfilled. The results were supported by previous theories, where engagement had a relationship with one’s level of well-being [45,46,49,51] although did not directly reduce stress.

Despite the substantial evidence for the importance of parental support for doctoral students [9,19,86], our study did not find similar evidence among those students majoring in mathematics education. However, parental support was proven to be the most significant factor in reducing their stress levels. According to the mathematics education doctoral students in Indonesia, emotional support from parents was only likely to reduce stress levels when they encountered problems in learning. This is aligned with the Asian culture, where children are always eager for positive feedback from their parents on their achievements [87]. Our study also found that reducing stress levels can increase the well-being of mathematics education doctoral students. In addition, a high level of well-being will increase the academic performance of mathematics education doctoral students. However, compared with parental support, other individual factors such as engagement, internal motivation, and well-being were more important in achieving academic performance when pursuing a doctoral degree. Although parental support did not significantly improve the academic performance of these students, the connection with parents was still important in reducing their stress levels and motivating their learning [53]. This improvement indirectly facilitates them to achieve the best performance while pursuing a doctoral program.

The significant positive effect of support from advisors and faculties on academic performance suggested by past studies was echoed in our study on mathematics education doctoral students. Past research has emphasized the role of the supervisor (or advisor) in doctoral students’ development as a researcher [2,20], as well as how faculty motivated them differently according to doctoral students’ needs [17,88]. The student–supervisor relationship becomes more critical in students’ success in doctoral programs because most Indonesians believe that supervisors are often able to determine students’ success at the institutional level. They also believe that the faculty members are influential academically and emotionally. Furthermore, Indonesian students usually consider their teachers (or supervisors at the doctoral level) as the only people who can provide advice and support when they meet difficulties during doctoral study. In this case, students could mostly become emotionally satisfied, gain valuable experience, and develop academically when their supervisors maximally support them. Several previous studies indicated that other faculty members’ support was another main key to learning success [57,89,90]. In this context, universities need to provide training for supervisors and other teaching staff regarding how to effectively support doctoral students’ studies and emotions. In addition, it was suggested that the supervisors could encourage doctoral students to collaborate during research projects and that the improvement of their cooperation skills significantly affected their academic performance. The ability to collaborate is an important skill in the 21st century [91,92,93]. A successful cooperative research project is likely to lead to better academic outputs [94,95]. These results emphasized the maintenance of communication and collaboration among students and the faculty. This should also be accompanied by listening to the problems of the students and releasing their mental problems to improve their academic achievement.

Facilitating conditions, namely the institutional environment where mathematics education doctoral students conduct research, did not directly affect their academic performance. This was due to the common inadequacy of these conditions in improving educational skills and efficiency in the local universities. In this case, the students were unable to effectively use existing facilities, collaborate, discuss with supervisors, or participate in seminars and training. However, the mathematics education doctoral students in Indonesia believed that facilitating conditions increased their level of well-being. This is also verified in other studies that show that facilitating conditions can also be a strong positive factor in increasing the well-being of doctoral students [49,96]. Furthermore, institutions with advanced facilitating conditions would receive more funding resources, which are important to students’ academic progress. Moreover, each supervisor is supposed to determine the forms of assistance, facilities, and support according to the current doctoral students [97].

## 6. Conclusions

By using the PLS-SEM approach, this study revealed that the academic performance of mathematics education doctoral students was affected by individual, parental, and institutional factors in a complex mechanism. In this study, an empirical model was developed to predict the significant factors affecting the academic performance of mathematics education doctoral students in Indonesia. Examination of the results of the empirical model led to the verification of student engagement, teacher support, and well-being as the significant variables directly influencing the academic performance of these students. Student engagement, teacher support, and stress level also significantly affected their academic performance in an indirect way. Therefore, the results obtained contributed to the present understanding of doctoral students’ academic performance. In this case, each factor and the relationship between these factors should be further evaluated and demonstrated in other contexts. The results also provided important implications to parents, lecturers, doctoral supervisors, and policymakers regarding the improvement of doctoral education quality.

## 7. Overall Contributions

### 7.1. Theoretical Implications

This study was inspired by several limitations of the previous studies on factors significantly affecting the academic performance of doctoral students: (1) an oversimplified conceptualization of doctoral students’ academic performance; (2) limited knowledge of how different factors can contribute to doctoral students’ academic performance together; (3) little attention to doctoral students in developing countries and who major in a specific specialization field in education. Therefore, several theoretical contributions are provided to research students’ academic performance at the higher education level.

Firstly, we developed an empirical model capable of explaining how individual, institutional, and parental factors can affect doctoral students’ academic success. This model distinguished that five independent factors (namely, fear of delay, student engagement, parental support, teacher support, and facilitating condition) can significantly influence two intermediated factors (namely stress level and well-being), and further influence the academic performance of mathematics education doctoral students. The results obtained can enlighten how to improve students’ academic performance in doctoral programs, especially in the major of mathematics education. This output is capable of filling the research gap in the previous analysis of doctoral students’ performance, where factors were examined separately [65,98].

Secondly, a new technique, namely the PLS-SEM approach, was proven valid to predict doctoral students’ academic performance. This was different from previous studies, where educational achievement was mostly investigated through the pretest-posttest paradigm and CB-SEM approach, or at the K-12 level.

Thirdly, this study contributes to the limited evidence of the factors affecting the academic performance of doctoral students in developing countries, namely Indonesia. In this specific educational and cultural context, student engagement, teacher support, and well-being are the main factors directly influencing doctoral students’ academic achievement.

### 7.2. Practical Implications

Based on the results, practical implications are provided to institutions, supervisors, and parents regarding the improvement of doctoral students’ academic performance. The results show that the success of doctoral students is inseparable from the role of parents, lecturers, and student’s level of engagement. Therefore, collaboration is needed among these motivators, to reduce stress levels, improve well-being, and support doctoral students’ academic achievement. The campus should develop enhancement programs for the well-being of doctoral students, leading to the mindset that the acquisition of a doctoral degree is the appropriate step. For faculty in the institution and doctoral students’ supervisors, many suggestions regarding the improvement of students’ well-being and engagement were provided, including the following: (1) checking students’ mental health status regularly, (2) maintaining enthusiasm for learning but with regular breaks, (3) improving connections and mutual support among doctoral students, (4) providing sufficient resources and in-time support to doctoral students in their academic journey, and (5) improving the curriculum, and training according to students’ needs.

From the results, parental support reduced the stress level of doctoral students. This is very important information for parents, whose assistance is critical to reduce the stress of doctoral students, even though most of them are over 25 years old and married. For parents of doctoral students, it is of great importance to understand the students’ situations and provide emotional support for them to continue their academic career. Faculty members and supervisors also need more adequate knowledge of the family background of each student, and to cooperate with their family to support the doctoral student.

## 8. Limitations and Future Directions

The limitations of this study lie in the following aspects. Firstly, the conclusions are based on a voluntary sample of mathematics education doctoral students in Indonesia. This limited sample leads to limited generalizability of conclusions in other higher education systems in other countries. Secondly, the study solely concentrated on the factors at the individual, institutional, and parental levels, implying the need for subsequent investigation of more variables, such as support from friends or wider learning communities. For instance, Grevholm et al. [99] studied a group of doctoral students, supervisors, scholars from other universities, and policymakers, indicating the effectiveness of interactions among them in improving students’ achievement. A more detailed analysis of factors at each different level is worthwhile by studying successful cases of doctoral education in mathematics education around the world. Thirdly, a quantitative paradigm has dominated the analysis. This leads to the need for rich qualitative knowledge of the nuanced mechanisms by which these factors can contribute to the academic progress of mathematics education doctoral students.

## Figures and Tables

**Table 1:** Hypotheses in this study.

Code	Hypothesis
H1	Fear of delay positively influences doctoral students’ stress level.
H2	Fear of delay negatively affects doctoral students’ academic performance.
H3	Student engagement negatively impacts the stress level of doctoral students.
H4	Student engagement positively influences doctoral students’ well-being.
H5	Student engagement positively influences doctoral students’ academic performance.
H6	Parental support negatively affects doctoral students’ stress level.
H7	Parental support positively impacts doctoral students’ academic performance.
H8	Teacher support positively influences doctoral students’ well-being.
H9	Teacher support positively affects doctoral students’ academic performance.
H10	Facilitating conditions positively impact doctoral students’ well-being.
H11	Facilitating conditions positively influence doctoral students’ academic performance.
H12	Stress level negatively affects doctoral students’ academic performance.
H13	Stress level negatively affects doctoral students’ well-being.
H14	Well-being positively impacts doctoral students’ academic performance.

**Table 2:** Demographic information of participants.

Demographics		N	Percentage
Gender	Male	59	40.14%
	Female	88	59.86%
Age	20–24 years old	41	27.89%
	25–29 years old	86	58.50%
	30 years old and above	20	13.61%
Academic year	First	23	15.65%
	Second	41	27.89%
	Third	52	35.37%
	Fourth year above	31	21.08%
Marital status	Married	91	61.90%
	Not yet Married	56	38.10%
Job status	Not Yet Working	119	80.95%
	Already Working	28	19.05%

**Table 3:** Outer loadings, reliability, and convergent validity.

Constructs	Indicator	Outer Loadings	Cronbach’s Alpha	Composite Reliability	Average Variance Extracted (AVE)
Fear of Delay	FOD1: Delay in graduation	0.881	0.727	0.728	0.786
	FOD2: Dropout after the delay	0.892			
Student Engagement	SE1: Focus on learning	0.846	0.786	0.791	0.699
	SE2: Efforts in learning	0.841			
	SE3: Retention in learning	0.821			
Parental Support	PS2: Emotional support	0.969	0.932	0.933	0.936
	PS3: Motivational support	0.966			
Teacher Support	TS1: Respect for other people’s ideas	0.818	0.877	0.886	0.669
	TS2: Fairness to students	0.797			
	TS3: Encouraging collaborations	0.868			
	TS4: Emotional support	0.809			
	TS5: Support for student decisions	0.796			
Facilitating Conditions	FC1: Necessary conditions	0.747	0.600	0.600	0.556
	FC2: Helpful people	0.765			
	FC3: Training and seminars	0.724			
Stress Level	SL1: Emotional depression	0.722	0.840	0.861	0.600
	SL2: Exhausted feelings	0.778			
	SL3: Stressful feelings	0.801			
	SL4: Lost enthusiasm	0.804			
	SL5: Thoughts about dropout	0.765			
Well-being	WB1: Wide connections	0.808	0.854	0.859	0.695
	WB2: New experiences and skills	0.819			
	WB3: Confident feelings	0.873			
	WB4: Enjoyable learning	0.834			
Academic Performance	AP1: Course grades	0.651	0.811	0.823	0.571
	AP2: Career plan	0.817			
	AP3: Paper writing	0.81			
	AP4: Research presentation	0.694			
	AP5: Critical thinking	0.792			

**Table 4:** Results of the Fornell–Larcker test for assessing discriminant validity.

	Academic Performance	Facilitating Conditions	Fear of Delay	Parental Support	Stress Level	Student Engagement	Teacher Support	Well−Being
Academic Performance	**0.756**							
Facilitating Conditions	0.524	**0.746**						
Fear of Delay	−0.148	−0.234	**0.886**					
Parental Support	0.308	0.309	−0.25	**0.968**				
Stress Level	−0.269	−0.272	0.501	−0.278	**0.775**			
Student Engagement	0.659	0.599	−0.158	0.289	−0.26	**0.836**		
Teacher Support	0.687	0.522	−0.075	0.424	−0.216	0.518	**0.818**	
Well−being	0.711	0.611	−0.15	0.326	−0.368	0.683	0.583	**0.834**

**Table 5:** Results of the HTMT test for assessing discriminant validity.

	Academic Performance	Facilitating Conditions	Fear of Delay	Parental Support	Stress Level	Student Engagement	Teacher Support	Well-Being
Academic Performance								
Facilitating Conditions	0.738							
Fear of Delay	0.204	0.358						
Parental Support	0.347	0.416	0.304					
Stress Level	0.296	0.391	0.642	0.322				
Student Engagement	0.809	0.864	0.207	0.324	0.27			
Teacher Support	0.798	0.729	0.128	0.468	0.239	0.619		
Well-being	0.828	0.834	0.18	0.362	0.392	0.819	0.668	

**Table 6:** Variance inflation factor value for all constructs.

	Academic Performance	Facilitating Conditions	Fear of Delay	Parental Support	Stress Level	Student Engagement	Teacher Support	Well-Being
Academic Performance								
Facilitating Conditions	1.914							1.753
Fear of Delay	1.421				1.076			
Parental Support	1.316				1.145			
Stress Level	1.528							1.101
Student Engagement	2.121				1.1			1.733
Teacher Support	1.818							1.517
Well-being	2.481							

**Table 7:** Results of the initial hypothesis test.

Relationships	Path Coefficients(β)	Sample Mean	Standard Deviation	T-Statistics	*p*-Values	Result
H1: Fear of Delay −> Stress Level	0.446	0.451	0.063	7.067	0	Supported
H2: Fear of Delay −> Academic Performance	−0.051	−0.053	0.064	0.803	0.422	Not Supported
H3: Student Engagement −> Stress Level	−0.154	−0.159	0.081	1.913	0.056	Not Supported
H4: Student Engagement −> Well−being	0.396	0.39	0.096	4.138	0	Supported
H5: Student Engagement −> Academic Performance	0.249	0.242	0.071	3.516	0	Supported
H6: Parental Support −> Stress Level	−0.122	−0.122	0.059	2.058	0.04	Supported
H7: Parental Support −> Academic Performance	−0.04	−0.043	0.075	0.537	0.592	Not Supported
H8: Teacher Support −> Well−being	0.235	0.239	0.073	3.228	0.001	Supported
H9: Teacher Support −> Academic Performance	0.392	0.396	0.074	5.335	0	Supported
H10: Facilitating Conditions −> Well−being	0.208	0.212	0.097	2.141	0.032	Supported
H11: Facilitating Conditions −> Academic Performance	−0.036	−0.032	0.073	0.499	0.617	Not Supported
H12: Stress Level −> Academic Performance	0.011	0.012	0.066	0.169	0.866	Not Supported
H13: Stress Level −> Well−being	−0.157	−0.159	0.054	2.939	0.003	Supported
H14: Well−being −> Academic Performance	0.344	0.345	0.079	4.337	0	Supported

**Table 8:** Results of indirect effect analysis.

Relationships	Path Coefficients (β)	Sample Mean	Standard Deviation	T-Statistics	*p*-Values	Result
Student Engagement −> Stress Level −> Academic Performance	−0.002	−0.002	0.012	0.147	0.883	Not Supported
Fear of Delay −> Stress Level −> Academic Performance	0.005	0.006	0.031	0.163	0.871	Not Supported
Student Engagement −> Stress Level −> Well−being	0.024	0.025	0.016	1.543	0.123	Not Supported
Student Engagement −> Stress Level −> Well−being −> Academic Performance	0.008	0.008	0.006	1.5	0.134	Not Supported
Student Engagement −> Well−being −> Academic Performance	0.136	0.136	0.05	2.704	0.007	Supported
Parental Support −> Stress Level −> Well−being −> Academic Performance	0.007	0.007	0.004	1.61	0.107	Not Supported
Parental Support −> Stress Level −> Academic Performance	−0.001	0	0.009	0.155	0.877	Not Supported
Teacher Support −> Well−being −> Academic Performance	0.081	0.081	0.029	2.824	0.005	Supported
Stress Level −> Well−being −> Academic Performance	−0.054	−0.055	0.022	2.43	0.015	Supported

## Data Availability

The data in this study can be provided upon request by sending an e-mail to the corresponding author.

## Appendix A

**Table A1:** Measurement items in the questionnaire.

Constructs	Code	Indonesian Version	English Version	References
Fear of delay	FOD1	Saya takut saya tidak dapat lulus tepat waktu 3 tahun	I’m afraid I can’t graduate in 3 years	
	FOD2	Saya takut saya tidak dapat menyelesaikan doctoral saya sampai 7 tahun yang menyebabkan saya harus drop out	I’m afraid that I won’t be able to finish my doctoral education until 7 years which will cause me to drop out	
Student engagement	SE1	Saya sangat focus pada Pendidikan doctoral saya	I am very focused on my doctoral education	[100]
	SE2	Saya pikir saya sudah melakukan yang terbaik during belajar doctoral saat ini	I think I did my best during my doctoral education	
	SE3	Saya tidak mempunyai planning untuk drop out dari doctoral saya	I have no plans to drop out of my doctoral education	
	SE4	Overall saya menikmati kuliah doctoral saya saat ini	Overall I am enjoying my current doctoral course	
Parental support	PS1	Orang tua saya memenuhi kebutuhan materi doctoral saya	My parents provide for my doctoral material needs	[19]
	PS2	orang tua saya selalu mensupport saya secara emosional saat saya menempuh doctoral	My parents have always supported me emotionally while pursuing my doctoral education	
	PS3	Overall, orang tua saya mendorong saya untuk meraih pencapaian tertinggi saat saya doctoral	Overall, my parents encouraged me to achieve the highest achievement during my doctoral education	
Teacher support	TS1	Dosen kami menginginkan seluruh mahasiswa respek terhadap ide orang lain	Our lecturers want all students to respect other people’s ideas	[41,101]
	TS2	Dosen kami adil terhadap seluruh mahasiswa	Our lecturers are fair to all students	
	TS3	Dosen kami mendorong mahasiswa bekerjasama	Our lecturers encourage students to work together	
	TS4	Dosen kami mengerti perasaan dan situasi setiap mahasiswa	Our lecturers understand the feelings and situations of each student	
	TS5	Dosen kami mensupport setiap ide dan keputusan kami	Our lecturers support our every idea and decision	
Facilitating conditions	FC1	Saya mempunyai kondisi yang memungkinkan untuk menempuh doctoral	I have the conditions necessary to pursue a doctorate	[102]
	FC2	Ketika saya mempunyai kesulitan saat menempuh doctoral saya tahu harus mencari siapa	When I have difficulties while pursuing my doctorate, I know who to look for	
	FC3	Kampus mempunyai banyak pelatihan dan seminar untuk meningkatkan academic performance mahasiswa	The campus has a lot of training and seminars to improve student academic performance	
Stress Level	SL1	Tugas dan tanggung jawab doctoral membuat saya Lelah secara mosional	Doctoral duties and responsibilities make me emotionally depressed	[103]
	SL2	Saya merasa kelelahan sepanjang saya menempuh doctoral saya	I felt exhausted throughout my doctoral education	
	SL3	Pendidikan doctoral membuat saya stress	Doctoral education makes me stressed	
	SL4	Saya kehilangan antusias belajar saya saat sudah menempuh doctoral	I lost my enthusiasm for learning when I took my doctorate	
	SL5	Saya mempunyai pemikiran untuk drop out dari doctoral saya	I have thoughts of doctoral education dropping out	
Well-being	WB1	Doctoral membuat saya mempunyai relasi yang luas	Doctorate made me have wide relations	[104]
	WB2	DOCTORAL membuat saya dapat memiliki banyak pengalaman dan skill baru	Doctorate allows me to have a lot of new experiences and skills	
	WB3	Secara keseluruhan, saya merasa percaya diri dan positif saat saya menempuh doctoral	Overall, I feel confident and positive while pursuing my doctoral degree	
	WB4	Saya enjoy merencanakan pengetahuan apa yang akan saya pelajari saat doctoral dan bagaimana cara mewujudkannya	I enjoy planning what knowledge to learn during my doctorate and how to make it happen	
Academic performance	AP1	Saya mendapatkan nilai yang memuaskan untuk setiap mata kuliah selama doctoral	I got satisfactory grades for every course during my doctoral degree	[105]
	AP2	Tidak sedikit ilmu yang saya dapatkan untuk masa depan saya saat saya menempuh doctoral	I got a lot of knowledge for my future while studying for my doctoral degree	
	AP3	Kemampuan saya dalam menulis paper meningkat selama doctoral	My ability to write papers increased during my doctorate	
	AP4	Kemampuan saya untuk melakukan presentasi di conference nasional atau international meningkat selama saya menempuh Pendidikan doctoral	My ability to make presentations at national or international conferences increased during my doctoral education	
	AP5	Saya merasa kemampuan berpikir kritis saya meningkat saat saya menempuh doctoral	I feel that my critical thinking skills have increased while pursuing my doctoral degree	
